# Advances in Modeling the Immune Microenvironment of Colorectal Cancer

**DOI:** 10.3389/fimmu.2020.614300

**Published:** 2021-02-10

**Authors:** Paul Sukwoo Yoon, Nuala Del Piccolo, Venktesh S. Shirure, Yushuan Peng, Amanda Kirane, Robert J. Canter, Ryan C. Fields, Steven C. George, Sepideh Gholami

**Affiliations:** ^1^ Department of Surgery, University of California, Davis, Sacramento, CA, United States; ^2^ Department of Biomedical Engineering, University of California, Davis, Davis, CA, United States; ^3^ Department of Surgery, The Alvin J. Siteman Cancer Center, Washington University School of Medicine, St. Louis, MO, United States

**Keywords:** colorectal cancer, tumor microenvironment, cancer immunology, tissue engineering, organ-on-a-chip (OOC)

## Abstract

Colorectal cancer (CRC) is the third most common cancer and second leading cause of cancer-related death in the US. CRC frequently metastasizes to the liver and these patients have a particularly poor prognosis. The infiltration of immune cells into CRC tumors and liver metastases accurately predicts disease progression and patient survival. Despite the evident influence of immune cells in the CRC tumor microenvironment (TME), efforts to identify immunotherapies for CRC patients have been limited. Here, we argue that preclinical model systems that recapitulate key features of the tumor microenvironment—including tumor, stromal, and immune cells; the extracellular matrix; and the vasculature—are crucial for studies of immunity in the CRC TME and the utility of immunotherapies for CRC patients. We briefly review the discoveries, advantages, and disadvantages of current *in vitro* and *in vivo* model systems, including 2D cell culture models, 3D culture systems, murine models, and organ-on-a-chip technologies.

## Introduction

In the US, colorectal cancer (CRC) is the third most common cancer and second leading cause of cancer-related death (1). CRC is largely asymptomatic until it has progressed to advanced stages (2), with 5 year survival rates of 90% and 14% for localized and metastatic cases, respectively (1). Population-wide screening campaigns in the last two decades have led to earlier diagnoses and boosted the overall 5 year survival rate to ~65% (1). Due to anatomical proximity, CRC often metastasizes to the liver: 20%–25% of patients present with colorectal liver metastases (CRLM) at initial diagnosis and 50-60% of CRC patients will develop CRLM at some point (2–4). Hepatectomy is currently the best course of action for CRLM patients, offering a 5 year survival rate of up to 60% (3–5). Unfortunately, only 20%–25% of CRLM patients are eligible for resection at time of diagnosis, leaving a large majority of patients to succumb to progressive metastatic cancer (3, 5).

Recent research has demonstrated the role of immunity on CRC progression, prognosis, and response to therapy. For example, immune cell infiltration into tumors correlates with clinical outcomes: T cells (6–10), Tregs (11), and NK cells (10) in primary CRC or CRLM lesions correlate with better prognoses, while the presence of tumor-associated macrophages (TAMs) has been alternately associated with pro- (12, 13) and anti-tumor (13–15) effects. In 2006, Galon and colleagues introduced the ImmunoScore. This measure of the density of immune cells in the invasive margin and core of a lesion (9, 16–18) provides more accurate predictions of recurrence, overall survival, and disease-free survival than traditional TNM staging for both CRC (7, 16, 18) and CRLM patients (6, 8, 11, 16).

Based on ImmunoScore’s prognostic success, clinicians are actively pursuing immunotherapies for CRC and CRLM patients. Checkpoint blockade therapies have shown particular promise for mismatch repair deficient (dMMR)/microsatellite instability-high (MSI-H) CRC tumors (19–21). In a 2015 Phase II clinical trial, dMMR/MSI-H patients treated with pembrolizumab (PD-1 inhibitor) exhibited a 40% response rate and 78% 12-month progression free survival (22); the FDA approved this course of treatment in 2017 (19). More recent work has probed the utility of combining nivolumab (PD-1 inhibitor) with ipilimumab (CTLA-4 inhibitor) (23). Results from this Phase II trial are still maturing, but preliminary results suggest a response rate as high as 55% (19, 23). Unfortunately, only 15% of CRC tumors are classified as dMMR/MSI-H (19), and there are currently no immunotherapies available to the remaining 85% of CRC patients. Pre-clinical work addressing this gap is focused on adoptive cell therapies, vaccines, immunostimulatory cytokines, and combinations thereof, and early studies have produced promising results (19–21).

The development of more efficacious cancer therapeutics is hindered by the limitations of current preclinical model systems, which do not recapitulate the whole tumor microenvironment (TME) (**Table 1** **)** (24, 25). The TME is crucial for investigating tumor-immune cell crosstalk, modeling tumor heterogeneity within and between patients, recapitulating events in the metastatic cascade, and simulating responses to therapeutics (24–28). 2D *in vitro* models of cells growing in tissue culture plates lend themselves to the study of tumor growth and cell migration, but lack complex tissue features like the vasculature and extracellular matrix (ECM) (24, 25, 28–31). In 3D *in vitro* models, multiple cell types can be co-cultured in ECM scaffolds, enabling the study of cell-cell interactions and nutrient/waste transport over small distances; however, these models lack key biomechanical features of the TME, including vascular and interstitial perfusion (24, 25, 28–31). Animal models are capable of simulating the dynamic, multi-cellular/organ nature of the TME, but are expensive, difficult to manipulate, and limited in their ability to recreate human immunobiology (24, 32–34).

**Table 1:** Advances in modeling colorectal cancer.

Model	Application	Advantages	Disadvantages
**2D *In vitro***	• Adhesion • Gene expression • Drug screening	• Simple • Low cost • High throughput	• Low predictive power • Lack of native architecture • Loss of tumor heterogeneity
Culture plate			
Wound healing	• Migration		
**3D *In vitro***	• Proliferation • Migration • Gene expression • Drug screening	• Retain native tumor geometry • Cell-cell/ECM interaction • Tumor heterogeneity	• Avascular • High cost • Low scalability • Low reproducibility
Organoid/ Spheroid			
Co-culture	• Stromal crosstalk • Immune crosstalk		
***In vivo*** Patient-derived xenografts	• Proliferation • Migration • Invasion • Angiogenesis • Gene expression • Drug screening	• Tumor heterogeneity	• High cost • Laborious • Low predictive power • Immunocompromised • Limited metastasis
Humanized mice		• Tumor microenvironment • Tumor heterogeneity • Immunocompetent	• High cost • Laborious • Incomplete immune function • Engraftment difficulties
Genetically engineered mice		• Tumor microenvironment • Tumor heterogeneity • Immunocompetent • Natural disease progression	• High cost • Laborious • Time consuming
**Organ-on-a-chip**	• Proliferation Migration • Intravasation Extravasation • Invasion • Angiogenesis • Stromal crosstalk • Immune crosstalk • Gene expression • Drug screening	• Tumor microenvironment • Tumor heterogeneity • Vascular • Hydrodynamic properties • Biochemical gradient • Precise control • Easy visualization	• Lack of standardization • High cost • Laborious • Low reproducibility

**Figure 1:**
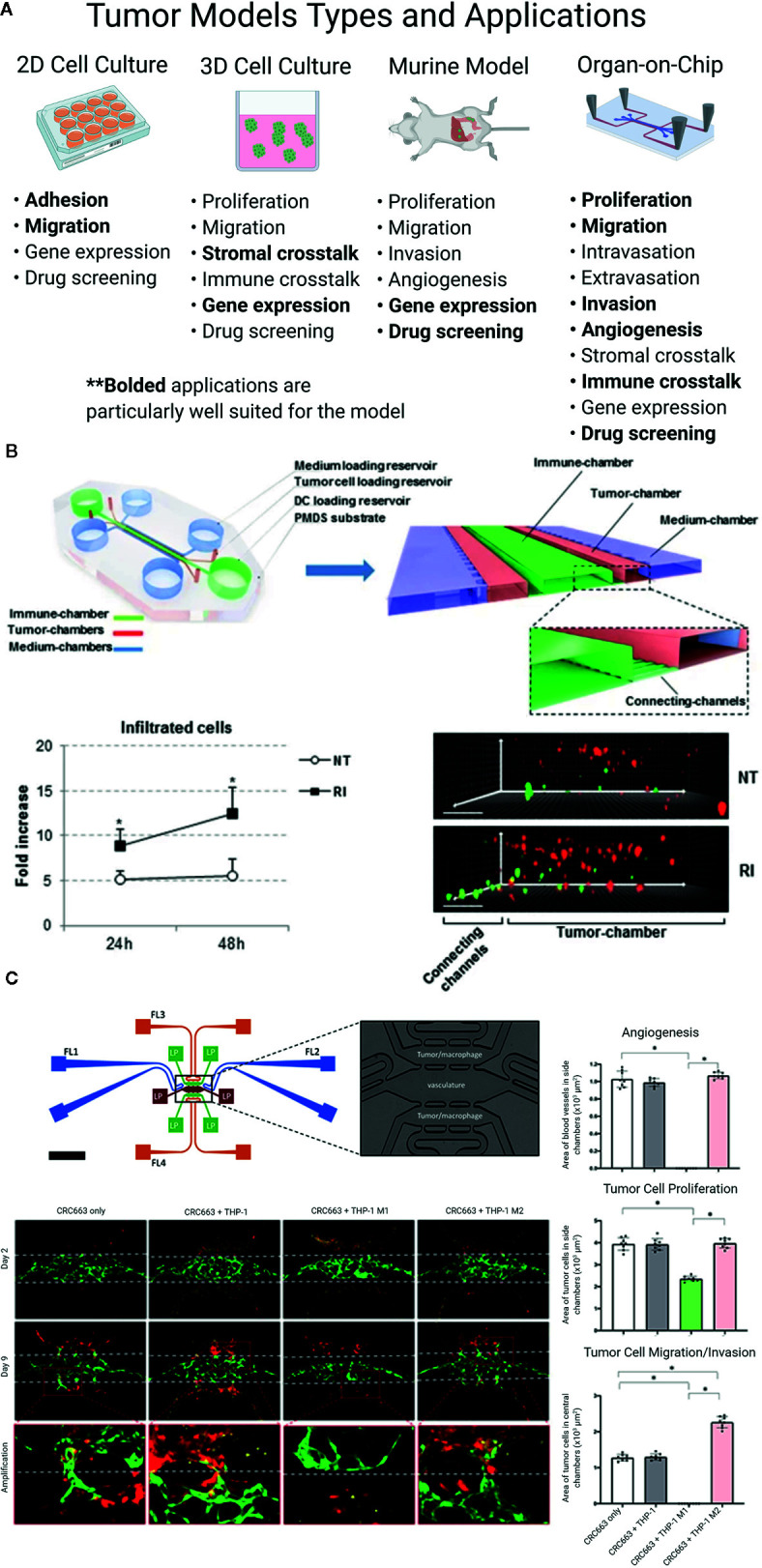
**(A)** Colorectal liver metastasis models. Bolded applications are particularly well suited for the model. Even in the same category of model, constituent models can vary greatly, based on design, method, and study goals. **(B, C)** Colorectal cancer (CRC) “organ-on-a-chip” (OOC) platforms can model the immune response to tumors. **(B)** CRC cells (red) and IFN-DCs (green) are cultured in an OOC device (see cartoon) to simulate immune crosstalk. IFN-DCs migrate towards and phagocytose CRC cells following treatment with interferon-α and romidepsin. Images have been adapted from Parlato et al’s 2017 *Scientific Reports* article (38). **(C)** M1 and M2 macrophages (red) cultured with CRC cell lines (not shown) in a vascularized OOC platform (vessels shown in green) display anti- and pro-tumor effects, respectively. Figure was originally published in Bi et al’s 2020 *Integrative Biology* report (39).

Model systems that combine tissue engineering with microfluidic technology represent a new frontier for the study of cancer development, progression, immunity, and metastasis. Dubbed “organ-on-a-chip” (OOC) systems, these models incorporate many features of the TME, including multiple cell types, matrix components, biochemical cues, spatiotemporal distribution of soluble mediators and oxygen, and perfusable vascular networks (24, 26, 35–37). Thus, OOC platforms offer great potential as a preclinical tool for precision therapy. This review will highlight recent advances in the utility of OOC devices to model immunity in the CRC/CRLM TME and compare this work with conventional model systems (**Figure 1A**).

## Two-Dimensional (2D) Cultures

Cell cultures in 2D (**Figure 1A**) are a standard and well-established model system because they are simple, inexpensive, and easy to manipulate, and enable imaging with high spatiotemporal resolution (**Table 1**) (24, 31). 2D cultures rely on cells adhering to a flat surface—generally a flask or plate—which does not reflect the natural 3D architecture of tissues or tumors. Furthermore, cells in 2D cultures receive relatively uniform and often excessive levels of oxygen, nutrients, and growth factors, compromising their ability to faithfully capture the *in vivo* TME (28–31). Despite these drawbacks, 2D experiments have revealed multiple mechanisms driving the behavior of epithelial sheets of cells (30) and epithelial-derived tumors like CRC and CRLM (40–50).

2D cultures are conducive to studies of tumor-immune cell crosstalk in the TME. For example, 2D *in vitro* systems have been used to examine the role of the CRC TME’s atypically high number of macrophages, a topic of active debate. These studies show that macrophages differentiate towards an M2-like phenotype in response to tumor cells or tumor cell-conditioned media (51–55) and migrate towards tumor cells (54, 55). TAMs in CRC have also been shown to modify the tumor cell response to chemotherapy (56, 57); support tumor cell proliferation, migration, and invasion (53–55); and limit tumor cell survival in a cell contact-dependent manner (13). Additionally, Yu and co-authors showed that mast cells migrate towards CRC tumor cell-conditioned media in a Transwell assay and that co-culture of mast and tumor cells increases tumor cell proliferation; the results of these 2D culture experiments were verified in a 3D spheroid model (58). Studies with primary patient samples have demonstrated i) an HLA-mediated T cell response to the survivin protein in CRC tumor cells (59), and ii) NK cell cytotoxicity directed against CRC tumor cells following immune cell activation or tumor cell priming (60).

## Three-Dimensional (3D) Cultures

3D cell cultures (**Figure 1A**) are comprised of cells distributed in synthetic or naturally-occurring scaffolds or hydrogels to mimic *in vivo* tissue architecture and can be cultured under static or perfused conditions (28–30). Compared to 2D cell culture systems, 3D *in vitro* systems more accurately model *in vivo* biochemical factor distribution and transport (28, 30); cell morphology, polarity, and gene expression (61–66); heterogeneity in cell types (62, 64, 67); and sensitivity to cancer therapeutics (61, 64, 67, 68). This accuracy is more pronounced under perfused culture conditions (61, 67–69) (**Table 1**). The challenges facing 3D cell culture systems include: i) uncertainty introduced by the underdefined, variable composition of popular scaffold materials (including the gold standard Matrigel); ii) the absence of vascular flow, which is responsible for cancer cell dissemination, trafficking of some immune cells, and delivery of therapeutics; iii) the inability to replicate the long-range interactions between tumors and other organs in the body that govern metastasis and the immune response (24, 29, 35, 70); and iv) limited reproducibility, scalability, and ease of use.

A handful of recent reports demonstrate the utility of 3D models systems for the study of immunity in the CRC TME. In a 2018 paper, Dijkstra et al. co-cultured organoids from dMMR CRC patients with autologous peripheral blood lymphocytes (71). In this novel culture system, the team generated patient-specific, cancer-reactive T cells from 4 of 8 patients, characterized the specificity of T cells for tumor versus healthy tissue, and measured the efficiency of T cell mediated tumor cell killing. A 2019 report by Courau and colleagues demonstrated that primary T and NK cells infiltrate into cell line-derived CRC spheroids, where they kill tumor cells and degrade the 3D structure of the spheroid, and that these effects can be enhanced by stimulating the immune response with IL-15 plus anti-NKG2D and/or anti-MICA/B antibodies (72). The authors also showed that stimulation of the immune response is necessary for infiltration of autologous T and NK cells into patient-matched CRC spheroids. Another recent study found that CAR-NK-92 cells engineered to recognize the universal antigen EPCAM, the neoantigen EGFRvIII, or the tumor-associated antigen FRIZZLED can identify and lyse cells in murine- and patient-derived normal colon and CRC organoids, but the effects are reduced by limited immune cell infiltration into organoids (73). Further, a 2019 report showed that primary CRC samples cultured under perfused conditions retained native tissue architecture, tumor cell density, and immune and stromal cell viability better than samples cultured under static conditions (69).

## *In Vivo* Models

*In vivo* models (**Figure 1A**) are integral tools in cancer research because they recapitulate several features of the TME not available in *in vitro* models, including vascular flow and communication between the tumor and distant organs (74–79). There are five types of mouse models of cancer: 1) xenograft, 2) allograft, 3) patient-derived xenograft (PDX), 4) humanized, and 5) genetically modified mouse (GEMM). Though murine models are labor intensive, expensive, low-throughput, and susceptible to cross-species incompatibilities, they have produced numerous insights into CRC response to drug treatment (76, 77, 80–87) and metastasis (85, 88–90) (**Table 1**).

Though transplant mouse models (xenograft, allograft, and PDX) accurately replicate the response to therapeutics (76, 77, 80–84), they struggle to retain the genetic and cellular features of native tumors (76, 79), recreate the metastatic cascade (with the possible exception of orthotopic transplant models) (89), and mimic the immune response to a tumor (note that xenograft and PDX models are both necessarily immunocompromised to enable inoculation with human cell lines and primary human tumor cells, respectively) (76, 79). Hence, humanized mouse models and GEMMs are more useful for studies of immunity in the TME. Humanized mice are generated by engrafting specific mouse strains with human leukocytes (hematopoietic stem cells or peripheral blood mononuclear cells). These mice produce a human immune response and are available commercially (91, 92), but sometimes suffer from xenoreactive complications and do not mount a full humoral immune response (74). In the context of CRC, humanized mouse models have been used to study tumor response to checkpoint blockade therapies (93, 94). In a 2015 report, humanized mice engrafted with a CRC cell line and treated with urelumab (CD137 inhibitor), nivolumab (PD-1 inhibitor), or a combination of the two demonstrated limited tumor growth and high infiltration of tumors by lymphocytes (93). Capasso and co-authors created humanized PDX mouse models by implanting patient-derived MSI-H or microsatellite stable (MSS) tumor cells and then treated the mice with nivolumab (PD-1 inhibitor) (94). Mice bearing MSI-H tumors showed high T cell infiltration into tumors and inhibited tumor growth compared to mice bearing MSS tumors; these results match clinical observations.

GEMMs are created by activating or deactivating specific genes using genome editing technology (75, 89, 95). These models retain a natural murine immune system; can simulate the natural development of CRC tumors from adenoma to carcinoma to metastasis (85, 88, 90); and can reproduce tumor response to therapy (75, 85–87, 95). Drawbacks to GEMMs include that they are time consuming and expensive to generate and characterize, and have a long time course of disease progression compared to other model systems (75, 89, 95). Tauriello and colleagues reported a set of GEMMs with mutations in one or more of the CRC-associated genes *Apc*, *Kras*, *Tgfbr2*, and *Trp53* (87); these models recreate many features of the human TME, including well-differentiated cancer cells, desmoplasia, and metastasis to the lung and liver. In subsequent experiments, the research team transplanted organoids from these CRC GEMMs into C57BL/6J mice to produce a model of advanced disease characterized by immune cell exclusion, increased TGFβ activity, and metastasis. Treatment with galunisertib (TGFBR1 inhibitor) reduced tumor growth and metastasis, increased immune cell infiltration and activation, and rendered tumors more responsive to anti-PD-L1 immunotherapy. Kostic et al. used a CRC GEMM model with a mutation in one copy of the *Apc* gene to explore the idea that the microbiome plays a role in CRC development (96). Mice were fed either a *Streptococcus* species or *Fusobacterium nucleatum*. The latter bacteria is found at higher levels in CRC tumor tissue than healthy colon tissue; indeed, mice fed *F. nucleatum* developed tumors more quickly and these tumors were infiltrated with high levels of pro-tumor immune cells, including myeloid-derived suppressor cells, granulocytes, neutrophils, TAMs, and M2-like macrophages. Some CRC cases are associated with colitis, a state of constant inflammation in the colon; colitis can be modeled in mice through treatment with azoxymethane and/or dextran sodium sulfate. Through comparisons of wild-type and knockout GEMM colitis mouse models, researchers have demonstrated the critical role of *p53* (97), IL-6 and Stat3 (98), TLR4 (99), *Pycard*, *Casp1*, and *Nlrp3* (100), and Nod1 (101) on tumor formation and growth; all of these factors are implicated in regulation of the immune response.

## Organ-on-a-Chip Models

OOC models (**Figure 1A**) utilize microfluidic technology and tissue engineering to mimic and monitor dynamic 3D tissue microenvironments, including epithelial barriers, parenchymal tissues, perfused microvasculature, multiple organ interactions, and the immune response (24, 37). An OOC platform consists of an interconnected series of 3D channels and chambers filled with cells suspended in hydrogels. The geometry of these channels and chambers can be precisely selected to match a variety of tissue architectures and mechanical forces, has a scale of tens to hundreds of microns, and is carved into an optically clear polymer using microfabrication or 3D printing (24, 102). Strengths of OOC systems include the ability to incorporate multiple human cell types at physiologically-relevant ratios; control hydrogel composition and spatial distribution; customize the physiochemical properties of the tissue microenvironment; and image tissues with high spatiotemporal resolution. Drawbacks of this emerging technology include difficulties transferring technology between labs, a lack of standardized benchmarks of success, and low-throughput experiments (**Table 1**).

Recent studies in CRC OOC models have successfully reproduced disease progression (103–106), immunity (38, 107–109), metastasis (110–112), and response to therapy (38, 103, 105–107, 110, 113). Biochemical gradients of growth factors, cytokines, and chemokines influence cell migration, tissue phenotype, and angiogenesis in the TME (114), and can be established, monitored, and perturbed using OOC technologies (24, 114–116). Emerging methods also enable the manipulation of hypoxia in OOC devices (117, 118); this property regulates gene transcription and alters physiological and pathological immunity (119, 120). Our group has also pioneered methods to vascularize tissues, including CRC, in OOC devices (39, 103, 106, 113, 121, 122). These blood vessel networks self-assemble when endothelial cells and stromal cells are mixed, suspended in hydrogels, and cultured under perfusion conditions. These microvasculature models mimic transport of cells, nutrients, waste, and therapies through tissues; and can be engineered from autologous cell sources.

OOC platforms can mimic the immune-tumor cell crosstalk found in the CRC TME. For example, Parlato et al. monitored the interactions between untreated and treated CRC tumor cells and interferon-α-conditioned dendritic cells (IFN-DCs)—a potential cancer therapeutic with the ability to uptake cancer antigens, stimulate a T cell response, and phagocytose tumor cells—in a 3D microfluidic model (**Figure 1B**). They observed that IFN-DCs preferentially migrate towards and phagocytose tumor cells that have been treated with interferon-α and romidepsin, thereby demonstrating the utility of the model for tracking immune-tumor cell interactions in real time and examining novel combination therapies. In a series of papers, an interdisciplinary team reported that patient- and murine-derived organotypic tumor spheroids cultured in microfluidic devices retain the tumor, stromal, and immune cell populations for multiple cancers, including CRC (107–109). The team also demonstrated that this model system recreates the tumor response to checkpoint blockade therapy more accurately than 3D *in vitro* systems and can be used to screen novel therapeutics for efficacy. A 2020 report from our group probed the role of M1 and M2 macrophages in the TME using a vascularized CRC OOC model (39) (**Figure 1C**). Our results showed that M1 macrophages inhibit angiogenesis and tumor cell growth and migration, while M2 macrophages have the reverse effect. Further, we showed that these outcomes are mediated by macrophage-derived soluble factors, suggesting new therapeutic targets and demonstrating the utility of the OOC platform to characterize the CRC TME.

## Future Directions

Improvement of CRC and CRLM patient outcomes requires the development of efficacious, targeted therapies. Immune-mediated therapeutic strategies are particularly promising but remain unrealized, which can be partially attributed to the inability of current *in vitro* and *in vivo* models to fully recapitulate immunity in the TME. 2D culture experiments provide an informative picture of tumor-immune cell crosstalk, but are limited in the number of cell types that can be examined simultaneously and cannot mimic *in vivo* transport of cells and secreted factors. 3D culture systems can support multiple cell types, mimic transport of biochemical factors through tumor tissue, and reproduce tumor response to immunotherapy, but lack the vascular supply necessary to mimic *in vivo* transport of immune cells to and through the tumor. Murine models have been critical to characterizing the immunobiology of the CRC TME, but these models struggle to accurately recapitulate metastasis; further, successful transition of therapeutics from murine studies to clinical practice remains quite limited. OOC platforms are capable of recapitulating the CRC TME, characterizing tumor-immune cell crosstalk, and mimicking patient-specific tumor response to therapy, but remain limited in their ability to model metastasis.

In contrast to the extensively characterized and utilized 2D culture, 3D culture, and mouse model systems, OOC platforms remain in early-stage development with untapped potential. Future work with OOC technology should focus on recreating colon-specific biological and physiochemical features of the primary CRC and metastatic CRLM TME. In particular, these models should seek to: i) incorporate tumor, stromal, and immune cells at the ratios found in the native TME; ii) mimic both MSS and MSI-H tumors; iii) utilize patient-specific cell sources; and iv) recreate the metastatic cascade by connecting CRC tissue models to liver tissue models using microfluidics. These advances in experimental modeling, especially when coupled with unforeseen progress, will produce additional knowledge regarding immunity in the CRC and CRLM TMEs and tumor response to immunotherapies, which may inform future clinical strategies and patient outcomes.

## Author Contributions

PSY, NDP, VSS, and YP performed the literature review. PSY and NDP wrote the manuscript. VSS, YP, AK, RJC, RCF, SCG, and SG critically reviewed and edited the manuscript. YP, VSS, and NDP created the figure. PSY compiled the table. RCF, SCG, and SG received the funding sources. All authors contributed to the article and approved the submitted version.

## Funding

This work is supported in part by grants from the National Institutes of Health (R21 CA223836, Fields and George), the Cancer Research Coordinating Committee (CRCC, University of California System, Gholami and George).

## Conflict of Interest

SCG is co-founder of Aracari Biosciences, a start-up company focused on the commercialization of vascularized OOC technology.

The remaining authors declare that the research was conducted in the absence of any commercial or financial relationships that could be construed as a potential conflict of interest.
